# 3T high-resolution magnetic resonance imaging, conventional ultrasonography and ultrasound biomicroscopy of the normal canine eye

**DOI:** 10.1186/s12917-021-03108-0

**Published:** 2022-02-10

**Authors:** Daniel Ivan, Stefanie Ohlerth, Henning Richter, Dagmar Verdino, Antonella Rampazzo, Simon Pot

**Affiliations:** 1 Clinic for Diagnostic Imaging, Department for Clinical Diagnostics and Services, Vetsuisse Faculty, University of Zurich, Zurich, Switzerland; 2 Veterinary Anesthesia Services International GmbH, Winterthur, Switzerland; 3 Ophthalmology Section, Equine Department, Vetsuisse Faculty, Zurich, Switzerland

**Keywords:** Dog, Eye, HR-MRI, MRI microscopy, Microcoil, 3 Tesla, Ultrasound, Ultrasound biomicroscopy

## Abstract

**Background:**

Advances in MRI coil technology and increased availability of high-field MRI in veterinary medicine enable the acquisition of images of increasingly high spatial resolution while preserving signal-to-noise ratio.The purpose of the present study was to compare 3T high-resolution magnetic resonance imaging (HR-MRI) with ultrasound (US) and ultrasound biomicroscopy (UBM) in the normal canine eye, to assess its potential to depict normal ocular anatomy.

**Results:**

HR-MRI was compared with US and UBM in 10 eyes from 10 healthy beagle dogs. Ocular structures (cornea, anterior chamber, iridocorneal angle, iris, lens, ciliary body, choroid, vitreous body, posterior wall of the eye, optic nerve and optic nerve sheath, extraocular muscles) were assessed subjectively and central corneal thickness (CCT), anterior chamber depth (ACD), aqueous depth (AQD), anteroposterior, mediolateral and dorsoventral lens diameter (APLD, MLLD, DVLD), anteroposterior diameter of the globe including and excluding the scleroretinal rim (APDSRR, APD), vitreous chamber depth (VCD) and optic nerve sheath diameter (ONSD) were measured in HR-MRI and in US. Optic nerve diameter (OND) was measured in HR-MRI. HR-MRI and UBM appearance of the anterior segment were subjectively compared. Detailed reference high-resolution MRI images of normal eyes of Beagle dogs are provided.

**Conclusions:**

HR-MRI allowed assessment of all structures identified with US and UBM. The MRI examinations were performed under general anesthesia with the addition of a neuromuscular blocking agent, while US and UBM examinations were performed in conscious animals. Visibility of the entire ocular wall, the lens, the structures caudal to the ciliary body and the optic nerve and its sheath was superior with HR-MRI. HR-MRI allowed the distinction of retina, choroid and sclera, and the delineation of structures not previously identified in canine eyes with MRI, including Tenon’s capsule and the sub-Tenon’s space.Plane selection was more accurate with HR-MRI compared to US. In general, the range of measurements was narrower for MRI than for US. CCT, AQD, APLD, MLLD, APD, APDSRR and ONSD differed significantly between HR-MRI and US, respectively (p = 0.005-0.027).Micro-MRI may be useful for the assessment of ocular pathologies in the future.

## Background

Imaging of the eye is used in conjunction with clinical ophthalmologic examinations, in particular in cases with obscured structures either due to their anatomical position or ongoing pathology. Conventional ultrasonography (US), ultrasound biomicroscopy (UBM) and magnetic resonance imaging (MRI) have been previously used in veterinary medicine to assess ocular and orbital pathology [1–6]. Advances in MRI coil technology and increased availability of high field MRI machines in veterinary medicine enable the acquisition of MRI images of higher spatial resolution than previously possible. Compared to standard MRI, high resolution magnetic resonance imaging (HR-MRI), also referred to as MRI microscopy, can achieve sub-millimeter resolution with good signal-to-noise ratio [7–10]. A thorough understanding of normal MRI appearance and artefacts is a prerequisite for recognizing pathological processes. Although the eyes of various species have successfully been examined with MRI ranging from 0.2 to 9.4T [5, 11–19], reference literature regarding the applications of HR-MRI to the canine eye is scarce [1]. The purpose of this study was to investigate the use of 3T microcoil HR-MRI and explore its potential to depict normal anatomy of the canine eye in comparison with US and UBM.

## Materials and methods

### Specimens

For this prospective research study, 10 healthy intact male experimental beagle dogs with a mean age of 4.7 years (range, 2- 8 years) and a mean body weight of 15.02 kg (range, 11.6 - 18.4 kg) were included. The study was carried out in compliance with the ARRIVE guidelines and the Swiss animal welfare act. The study was approved by the governmental authorities (animal permission number ZH273/16). All dogs were owned for research purposes by the Vetsuisse Faculty of the University of Zurich. The dogs were considered healthy based on the clinical examination performed by the anesthetist (DV), and on the results of blood chemistry and hematology analysis. Ophthalmological examination performed by a board-certified veterinary ophthalmologist (SP) identified no ocular abnormalities other than a healed eyelid laceration, a small papillomatous skin growth and a single small retinal fold in three separate dogs. Ten eyes (5 right and 5 left) of 10 dogs were examined using high resolution 3T MRI (HR-MRI), conventional ultrasound (US), and ultrasound biomicroscopy (UBM).

### Anesthesia and instrumentation of dogs

All dogs were fasted before examination, were premedicated with methadone (0.2 mg/kg, IV; Methadon Streuli^®^, Streuli Pharma AG, Uznach, Switzerland) and a catheter was placed in the cephalic vein. Preoxigenation was provided via a facemask and anesthesia was induced intravenously using lidocaine (2 mg/kg; Lidocain 2% Streuli^®^, Streuli Pharma AG, Uznach, Switzerland), ketamine (1.0 mg/kg; Ketnarkon^®^ 100 ad us. vet., Streuli Pharma AG, Uznach, Switzerland), midazolam (0.1 mg/kg; Midazolam Sintetica^®^, Sintetica S.A., Mendrisio, Switzerland), and propofol (3.0 to 5.3 mg/kg, to effect; Propofol 1% MCT Fresenius^®^, Fresenius Kabi AG, Oberdorf, Switzerland). After intubation, anesthesia was maintained with sevoflurane (Sevoflurane^®^, Abbott AG, Baar, Switzerland) administered to effect in an oxygen-air mixture. Mechanical ventilation was initiated and infusion of lactated Ringer’s solution (5mL/kg/h, IV) was administered throughout the procedure. Cardiovascular and respiratory variables including inspired and expired sevoflurane concentrations were monitored continuously and recorded by a multiparameter monitor. To limit eye movement during the MRI scan and for centralisation of the globe, a non-depolarizing muscle relaxant (Rocuronium Fresenius^®^ Fresenius Kabi AG, Oberdorf, Switzerland) was administered intravenously [20]; the initial dose was 0.4 mg/kg. Additional injections at a dose of 0.2 mg/kg were given every 20 minutes during the examination (2-3 times per procedure).

### Diagnostic imaging

**Fig. 1:**
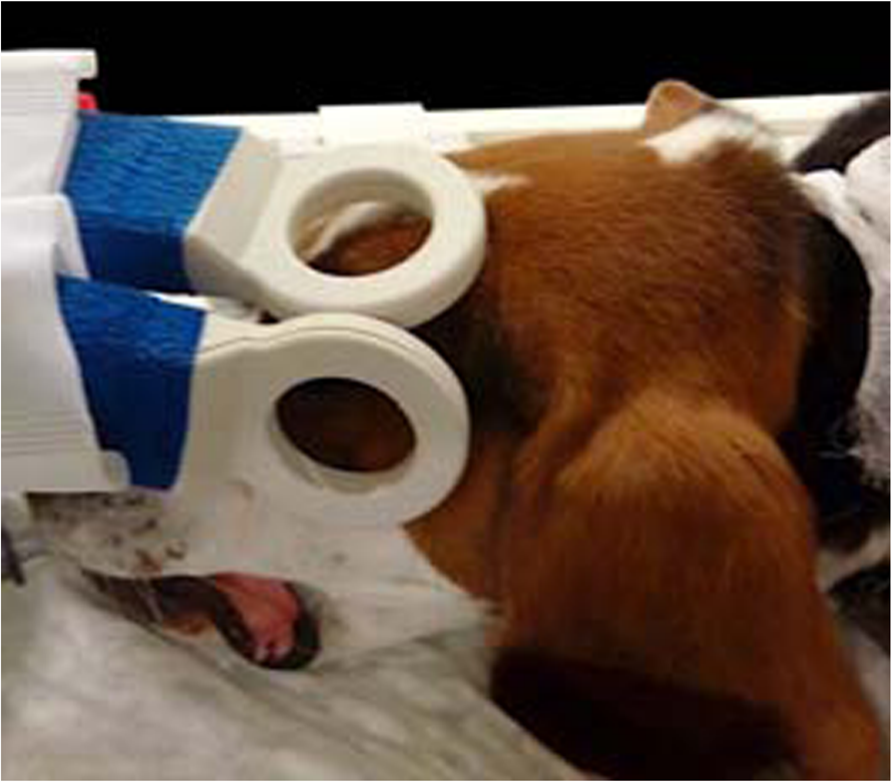
Microcoil placement: the coils are placed at a 90^∘^ angle in relation to each-other, parallel to B0 (main static magnetic field), as close as possible to the orbit

A high-field MRI scanner (Philips Ingenia 3T, Philips AG, Zurich, Switzerland) and experimental microscopy surface coils (Micro47, Philips Medical Systems) were used. The receive coil ensemble consisted of two two-channel, independent, parallel, receive circular loop coils with a diameter of 47 mm. The dogs were placed in sternal recumbency with the head oriented towards the gantry. The coils were placed as close as possible to the region of interest using a custom built holder with flexible arms, adhesive tape and straps. The coils were aligned with the bore axis (B0) and placed at approximately 90 degrees to each other, while also complying with the anatomy of the orbital region (Fig. 1).

Pre-contrast T2w images in sagittal and transverse, T2 SPAIR (SPectral Attenuated Inversion Recovery) images in dorsal and T1w images in dorsal planes were acquired. Contrast medium (Gadodiamid, Omniscan^®^, 0.3 mmol/kg, GE Healthcare AG, Glattbrugg, Switzerland) was applied manually, followed by a bolus of 5 ml saline. Subsequently, T1w post-contrast images in the transverse plane and T1SPAIR in 4 dogs and T1SPIR (Spectral Presaturation with Inversion Recovery) in 6 dogs in the dorsal plane were acquired. Positioning of the slices was adapted to the orientation of the axis of the eye globe (axis bulbi) for the sagittal and dorsal sequences and to the lens and tips of the ciliary processes for the transverse images. All sequences included 18 slices with a thickness of 1.5mm and an interslice gap of 0.1 mm. For four dogs, a field of view (FoV) of 80 ×80 mm with a matrix ranging between 880 ×880 to 1024 ×1024 pixels was used (90 ×90 *μ*m to 78 ×78 *μ*m in plane resolution). For the following 6 dogs the FoV was decreased to 40x40 mm with a matrix of 448 ×448 to 864 ×864 pixels, delivering an in plane resolution of 89 ×89 *μ*m to 46 ×46 *μ*m. The total acquisition time was 40 to 50 minutes per eye. Detailed scan parameters are listed in Table 1.

**Table 1:** Summarized MRI sequence parameters

Sequence	Plane	TR (ms)	TE (ms)	Matrix	NSA
T2w	sagittal plane	2000	101	448 ×448	4
T2-SPAIR^1^	dorsal	2000	93	864 ×864	3
T1w pre-contrast	dorsal transverse	16	597-602	512 ×512	3
T1-SPIR^2^ post-contrast	dorsal	16	690	448 ×448	5
T1w post-contrast	transverse	16	597	512 ×512	3

A total of 6 and 4 dogs were scanned using a FoV of 40x40mm and 80x80mm, respectively, and a slice thickness of 1.5 mm. ^1^T2-SPAIR - SPectral Attenuated Inversion Recovery, fat suppression technique combining features of Fat-Sat pulses and STIR (Short Tau Inversion Recovery) [23] ^2^T1-SPIR - Spectral Presaturation with Inversion Recovery, hybrid technique combining a fat-selective RF-pulse and spoiler gradient with nulling of the residual longitudinal fat magnetization through an inversion delay mechanism [23]

Conventional ultrasonography (US) was performed by a diagnostic imaging resident (DI) and a board certified radiologist (SO) using a 16 MHz broadband linear transducer (Aloka ProSound F75, Hitachi Medical Systems Europe, Zug, Switzerland). Animals were restrained manually and positioned in sternal recumbency. A local anesthetic (Oxybuprocain 0.4%, Novesin^®^, OmniVision AG) was applied to the eye and sterile transmission gel (Aquasonic^®^ 100, Parker Laboratories, Fairfield, USA) was applied to the cornea. While avoiding direct contact with or pressure on the cornea, transcorneal vertical and horizontal axial and oblique still images and loops were acquired [21]. Transverse sections of the eye globe and orbital space were acquired using a lateral (temporal) ultrasonographic window. Ultrasound biomicroscopy (UBM) images from the dorsotemporal quadrant of the eye were acquired by a board certified veterinary ophthalmologist (SP) using a 40 MHz mechanical transducer (Eyecubed I-3 System, version 4; Ellex, Innovative Imaging, Sacramento, CA, USA). Both paraxial and transverse still images were acquired with the probe placed at the limbus and oriented perpendicular to and parallel with the limbus, respectively.

## Image analysis

An open-source DICOM-viewer (Horos v4.0.0) and an image-analysis program [22] were used for reviewing MRI, US and UBM images, respectively. The images were reviewed by a diagnostic imaging resident (DI), a board-certified veterinary radiologist (SO) and two board-certified ophthalmologists (AR, SP) individually and in consensus.

### MRI

**Fig. 2:**
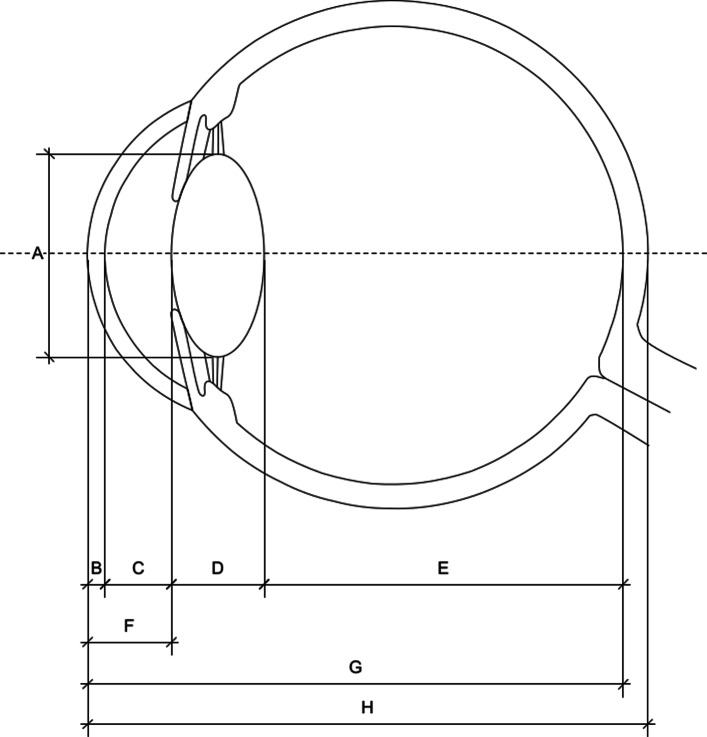
Schematic illustration of measurement parameters of the canine eye in the sagittal plane showing: A - Dorsoventral Lens Diameter (DVLD), B – Central Corneal Thickness (CCT), C - Aqueous Depth (AQD), D – Anteroposterior Lens Diameter (APLD), E – Vitreous Chamber Depth (VCD), F – Anterior Chamber Depth (ACD), G – Anteroposterior Depth of the eye excluding the scleroretinal Rim (APD), H - Anteroposterior Depth of the eye including the Scleroretinal Rim(APDSRR). The dotted line represents the axis of the bulbus oculi

Subjective assessment of the cornea, anterior and posterior chambers, lens, ciliary body, vitreous, the ocular wall, optic nerve and sheath complex, vascular structures, extraocular muscles and periocular structures was performed individually and in consensus on all available images. The visibility of anatomical structures, their signal intensity and contrast enhancement were evaluated. Parameters measured on MRI were the anterior corneal thickness (CCT), anterior chamber depth (ACD), aqueous depth (AQD), anterioposterior depth of the eye globe including the scleroretinal rim (APDSRR), anterioposterior depth of the eye globe excluding the scleroretinal rim (APD), mediolateral lens diameter (MLLD), dorsoventral lens diameter (DVLD), anteroposterior lens diameter (APLD), vitreous chamber depth (VCD), optic nerve sheath diameter (ONSD) and optic nerve diameter (OND). The latter could only be determined on MRI due to the contrast provided by the T2 hyperintense signal from the spinal fluid present in the optic nerve sheath. A graphic representation and descriptions of the measured parameters are listed in Fig. 2 and Table 2.

**Table 2:** Ocular parameter definitions

Parameter name	Definition
Central corneal thickness (CCT)	Distance between the two parallel hyperechoic / hyperintense lines representing the corneal epithelium and basement membrane on the outside and Descemet’s membrane and the corneal endothelium on the inside, in the mid-sagittal plane, along the axis of the globe [26].
Anterior chamber depth (ACD)	Distance between the central anterior corneal epithelium to the anterior lens capsule, along the axis of the globe [27].
Aqueous depth (AQD)	Distance between the corneal endothelium and the anterior surface of the lens, along the axis of the globe [27].
Anteroposterior Lens Diameter (APLD)	Distance between the anterior and posterior capsule of the lens, at the widest point, in the sagittal plane.
Mediolateral Lens Diameter (MLLD)	Distance between the equators of the lens, at the widest point, in the dorsal plane.
Dorsoventral Lens Diameter (DVLD)	Distance between the equators of the lens, at the widest point, in the sagittal plane.
Vitreous chamber depth (VCD)	Distance between the posterior lens capsule and the innermost surface of the retina, along the axis of the globe, in the sagittal plane.
Anteroposterior depth of the eye globe including the scleroretinal rim (APDSRR)	Distance between the central anterior corneal epithelium to the outermost surface of the sclera, along the axis of the globe, in the sagittal plane [3].
Anteroposterior depth of the eye globe excluding the scleroretinal rim (APD)	Distance between the central anterior corneal epithelium to the innermost surface of the retina, along the axis of the globe, in the sagittal plane [3].
Optic Nerve Sheath Diameter (ONSD)	Width of the optic nerve sheath, measured approx. 3mm caudal to the optic disc (dorsal plane) [28].
Optic Nerve Diameter (OND)	Width of the optic nerve, measured approx. 3mm caudal to the optic disc (dorsal plane, T2-SPAIR images)

All variables were measured on US and HR-MRI, with the exception of the OND. The optic nerve can not be delineated from its sheath via US

### US

Subjective assessment of grayscale ultrasound images [21, 24](22,27) was performed, evaluating visibility and ultrasonographic characterization of ocular and periocular structures. Central corneal thickness (CCT), anterior chamber depth (ACD), aqueous depth (AQD), anteroposterior depth of the eye globe including the scleroretinal rim (APDSRR), anteroposterior depth of the eye globe excluding the scleroretinal rim (APD), mediolateral lens diameter (MLLD), dorsoventral lens diameter (DVLD), anteroposterior lens diameter (APLD), vitreous chamber depth (VCD), and optic nerve sheath diameter (ONSD) were determined.

### UBM

Structures in the anterior segment including the cornea, sclera, iris, ciliary body, iridocorneal angle and ciliary cleft were assessed subjectively and compared to MRI, with a consensus evaluation of visibility and ultrasonographic characterization [25]. No measurements were performed in UBM due to the inherent low depth of penetration of this modality, the inability to image the entire globe and the resultant limitation in establishing reference points in relation to the axis of the globe when acquiring measurements.

## Statistical analysis

Statistical analysis was performed using a commercial software package (IBM SPSS Statistics for Mac, version 25.0, 16bit version, IBM Corp. Armonk, NY, USA). Descriptive analyses included median, minimum and maximum values and standard deviation (SD). Due to the small sample number, normal distribution of data was not assumed. Accordingly, non-parametric statistics (Wilcoxon rank sum test) were performed to analyze data within and between modalities. In this case, the null hypothesis *H*_0_ is that the two measurements have the same distribution with the same median. A *p*-value < 0.05 was considered significant to reject *H*_0_ and conclude that the distributions and medians differ.

## Results

### US

The structures of the globe, including cornea, anterior and posterior chambers, lens, iris, ciliary body, vitreous and posterior globe wall, as well as orbital structures, including the optic nerve and nerve sheath, extraocular muscles, vessels, fat and connective tissue, the surface of the orbital bone, and the lacrimal and zygomatic salivary glands could be identified with conventional ultrasound. Multiple cine loops acquired in the vertical, horizontal and oblique axial planes allowed the authors to obtain a complete overview of the ocular region. Images in the transverse plane could only be acquired from the temporal and dorsal quadrants with conventional US. Findings were consistent with previously described ultrasonographic characteristics of the canine eye [21].

### UBM

UBM allowed examination of the cornea, limbus, anterior parts of the sclera, iris, ciliary body, iridociliary angle, ciliary cleft, scleral venous plexus, anterior and posterior chambers and anterior lens capsule in paraxial, transverse and oblique planes, from the temporal and dorsal quadrants in all eyes. Acquiring UBM images from quadrants other than the central, temporal and dorsal quadrants can be very challenging in awake animals and was not attempted in this study. UBM findings were consistent with current literature [2].

### HR-MRI

The MRI images of all 10 eyes were of excellent diagnostic quality and provided good signal-to-noise ratio. In isolated cases, technique-related artefacts were noted, including movement artefacts (1 dog) and wrap-around artefacts (1 dog). Artefacts related to movement could be successfully reduced or eliminated in all dogs by re-injection of the muscle relaxant. Oversampling and changing the phase-encode were applied to reduce wrap-around artefacts. MRI allowed assessment of all components of the globe and orbit and all examined structures had similar appearance and signal behavior in all dogs. The following description of the various structures was based on an individual subjective assessment by three reviewers, followed by a consensus-building discussion.

### Ocular structures

**Fig. 3:**
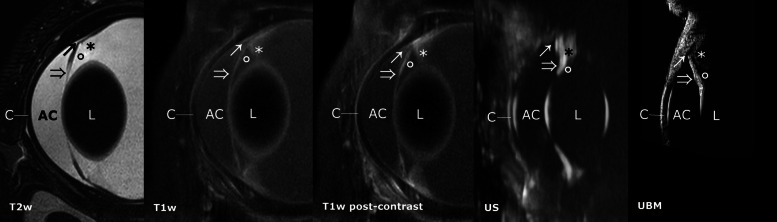
Comparative sagittal plane images of the anterior segment in HR-MRI T2w, T1w, T1w post-contrast, US and UBM. Cornea (C), note a discrete T2 hyperintense line coursing through the mid-portion of the corneal stroma on T2w HR-MRI images. Overview of the anterior chamber (AC) and posterior chamber (empty circle), especially well delineated T2w images due to the high contrast provided by the aqueous humor. Lens (L). Iris (empty arrow), iridocorneal angle (arrow), ciliary body (asterisk). Delineation of the iris and iridocorneal angle is superior with T2w HR-MRI compared to US and comparable to UBM. The ciliary body is not clearly delineated in the sagittal plane in T2w images and shows moderate contrast enhancement in T1w images

The cornea appeared as a hypointense layer, on sagittal T2w and dorsal T2SPIR images, outlined by the hyperintense tear film at the anterior margin and the hyperintense aqueous in the anterior chamber. A discrete, interrupted hyperintense line coursing through the mid-portion of the corneal stroma on T2w and T2SPAIR sagittal and dorsal images could be identified in consensus in 8 out of 10 dogs (Fig. 3). The cornea was homogeneously hypointense on T1w images and showed no contrast enhancement. The sclera was delineated as a smooth and hypointense layer in all sequences.

**Fig. 4:**
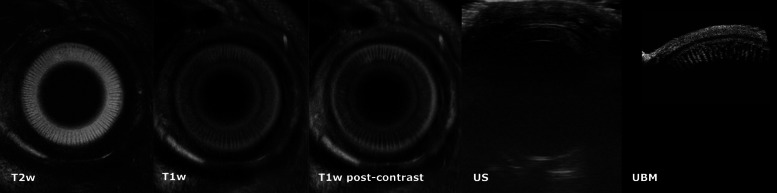
CComparative transverse HR-MRI, US and UBM images of the canine eye at the level of the corona ciliaris. HR-MRI T2w, T1w, T1w post-contrast, US, UBM. The alternating long and short ciliary processes are delineated as radially oriented hypointense linear structures on a hyperintense background on T2w images, as slightly hyperintense relative to the background in T1w images and with moderate contrast enhancement in T1w post-contrast images, respectively. The ciliary processes are poorly delineated in US. UBM allows clear delineation of the long and short ciliary processes, but only in the dorsotemporal quadrant of the eye

The ciliary crown (corona ciliaris) could be visualized as internally directed, radial, linear structures in the transverse plane on HR-MRI, appearing hypointense on a hyperintense background in T2w images, or as hyperintense linear structures with marked contrast enhancement in T1w images (Fig. 4 T2w, T1w and US), respectively). This was clearly delineated in 9 of the dogs and was less conspicuous in one. Visualization of all ciliary processes (both short and long) was dependent on minor variations in slice positioning.

The ciliary body appeared as a triangular shaped, hyperintense structure on a dark background on T1w pre-contrast, with moderate contrast enhancement on T1 SPIR and SPAIR post-contrast images, in all dogs. Conspicuity was very low on T2w and T2 SPAIR images, showing mildly heterogeneous signal intensity (Fig. 3). The iris was recognized as a thin, hypointense structure in dorsal and sagittal T2w images. In six of the dogs, the iris appeared two-layered. On T1w images, the iris was slightly less conspicuous, appearing moderately hyperintense, on a hypointense background, and its posterior aspect showed moderate contrast enhancement (Fig. 3).

**Fig. 5:**
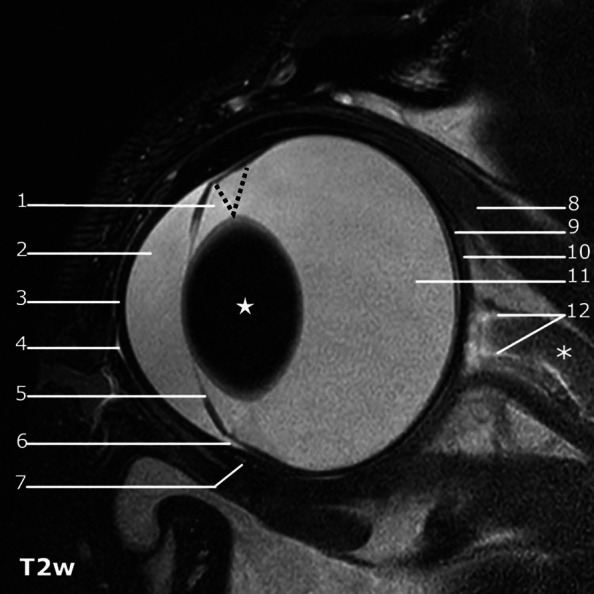
T2w sagittal image of the canine eye globe: 1) posterior chamber, 2) anterior chamber, 3) cornea, 4) hyperintense tear film on the surface of the cornea cornea, 5) iris, 6) iridocorneal angle, 7) scleral venous plexus iridocorneal angle, 8) dorsal rectus muscle, 9) sub-Tenon space, 10) Tenon capsule, 11) vitreous, 12) hyperintense CSF in the optic nerve sheath, optic nerve (asterisk), ciliary body (dotted line), lens (star)

At the base of the ciliary processes, punctiform areas which were hyperintense on T2 and showed marked contrast enhancement on T1w post-contrast images were identified as the scleral venous plexus and anterior ciliary arteries, in nine of the dogs (Fig. 5).

**Fig. 6:**
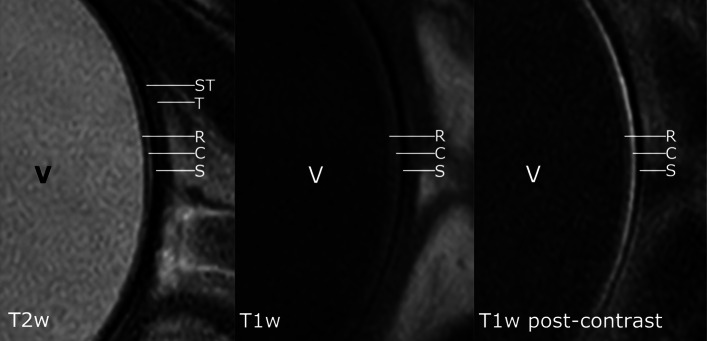
HR-MRI allows distinction of retina, choroid and sclera and identification of Tenon’s capsule and the sub-Tenon’s space. Detailed HR-MRI images of the posterior wall of the eye globe: HR-MRI T2w, T1w and T1w post-contrast. Vitreous (V), hyperintense in T2w images, hypointense in T1w. The sclera is hypointense in all sequences (S). Discrete laminar appearance of the choroid and retina, with alternating hyper- and hypointense layers, from the outside inward, in all sequences; the most outward of these layers shows marked contrast enhancement. Tenon’s capsule (T) and the sub-Tenon’s space (ST) can be clearly identified on T2w images

The ciliary cleft could be delineated on mid-dorsal and mid-sagittal T2w and T2 SPAIR images in all dogs. The ciliary cleft was considerably less conspicuous in T1w images (Fig. 3). The lens had a hypointense nucleus in all dogs, with an ill-defined T1 and T2 intermediately hyperintense outer portion of the cortex. The anterior lens capsule could be delineated in all dogs as a discrete hypointense line, tapering just posterior to the lens equator. The remaining portions of the posterior lens capsule could not be delineated (Fig. 3). The aqueous and the vitreous body were hyperintense on T2w images and showed low to intermediate signal intensity on T1w images, which was higher than the signal intensity of the lens nucleus (Fig. 3). Internal to the sclera (Fig. 6: S) and posterior to the equator, two discrete, distinct, laminar structures appearing as slightly hyperintense and hypointense bands (from outwards inwards), could be identified in the area of the choroid and retina in T1w images. These could clearly be delineated in 3 of the dogs by all reviewers and with variable confidence in the remaining dogs. After gadolinium administration, the outermost of these three layers enhanced markedly in six of the dogs, and variably in the remaining dogs. On T2w images, two corresponding distinct hyper- and hypointense layers, from the outside inwards, were delineated in the same region.

The optic disc could be delineated on sagittal and dorsal images in nine of the dogs as a focal interruption of the trilaminar structure of the posterior wall of the eye with low (lamina cribrosa/sclera) to intermediate (neuropil) signal intensity, directly internal to the optic nerve. This was inconsistently depicted in the transverse plane, due to slice positioning. Tenon’s capsule (vagina bulbi) could be identified on sagittal T2w images as a discrete hypointense sheath, at the caudal aspect of the globe (Fig. 5: No. 10; Fig. 6: T). Tenon’s capsule was separated from the outermost surface of the sclera by T2 hyperintense material (periscleral lymph space or sub-Tenon’s space) (Fig. 5: No. 11; Fig. 6: ST), fusing caudally with the optic nerve sheath and with the perimuscular fasciae.

### Ocular adnexa

Orbital structures can be further divided into intraconal (within the extraocular muscle cone) and extraconal (outside the extraocular muscle cone, but within the periorbita) compartments.

### Intraconal structures

**Fig. 7:**
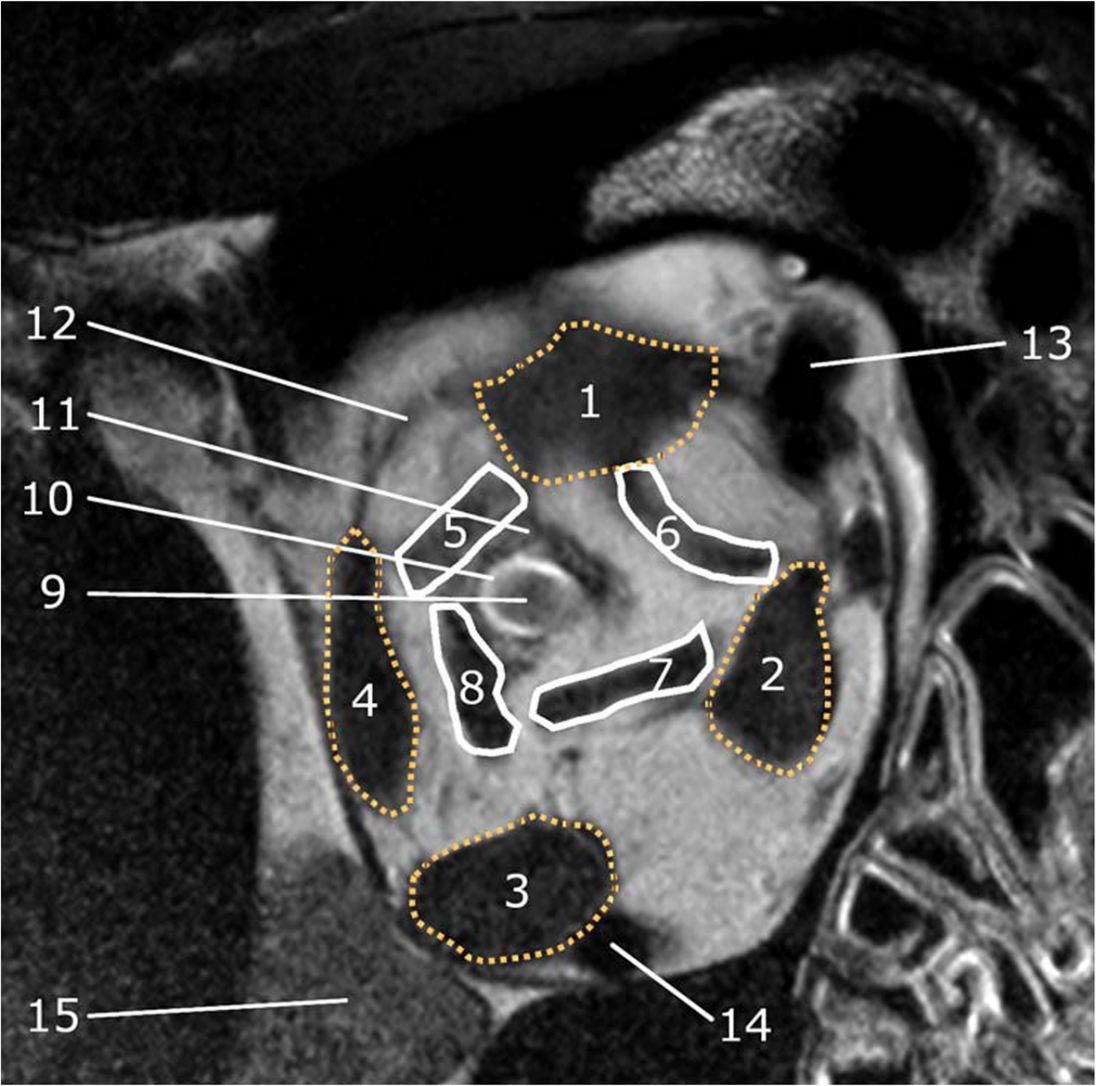
Transverse T2w HR-MRI image of the retrobulbar region Dotted lines: the rectus muscles 1) dorsal, 2) medial, 3) ventral and 4) lateral; surrounded by continuous lines are the retractor muscles (5, 6, 7 and 8), 9) optic nerve surrounded by 10) a rim of T2 hyperintense CSF within the optic nerve sheath, 11) optic nerve sheath, 12) portions of the periorbita, 13) dorsal external ophthalmic vein, 14) ventral ophthalmic vein, 15) Zygomatic gland, 16) periorbita

The optic nerve and its sheath could be best assessed on T2 SPAIR, followed by sagittal T2w images, due to the high contrast provided by the hyperintense layer of CSF that is contained in the subarachnoid space and the low signal intensity of the optic nerve itself (Fig.5: No. 12 and asterisk, respectively). In T1w images, the optic nerve was iso- or mildly hyperintense to the surrounding CSF. The course of the optic nerve was torturous and highly variable, depending on globe position. Assessment of the optic nerve was limited to the intraorbital segment due to the dimensions of the field of view with HR-MRI and the loss of signal from the intracanalicular region. Other nerves could not be confidently identified. The medial, lateral, ventral, and dorsal rectus muscles as well as the retractor muscles and dorsal and ventral oblique muscles could be identified in all dogs (Fig. 7, partially depicted in Fig. 5) and demonstrated low to intermediate T1 and T2 signal intensity and mild diffuse contrast enhancement [29]. Portions of the periorbita, appearing as thin hypointense membranes on the hyperintense background of the periorbital fat, spanning between the orbital muscles, could be identified confidently in 8 of the dogs, on transverse images.

### Extraconal structures

Meibomian glands, appearing as parallel, fusiform to tubular-shaped, T1 and T2 hyperintense structures, hypointense in T2SPAIR, could be identified in seven of the dogs, depending on slice positioning (not shown). The orbital ligament, spanning dorsolaterally between the zygomatic process of the frontal bone and the frontal process of the zygomatic bone was delineated as a thick T1 and T2 hypointense band in all dogs and was best seen in transverse images (not shown). No specific angiographic imaging sequences were used in this study, but the dorsal external ophthalmic vein, the ventral external ophthalmic vein and their anastomotic branch could be identified in all dogs (Fig. 7). Arteries were delineated inconsistently. Portions of the zygomatic gland were identified as a slightly lobulated structure with intermediate T1 and T2 signal intensity, at the ventral aspect of the orbit, in all dogs. No ocular abnormalities were observed in any of the dogs with the imaging techniques used. A retinal fold identified ophthalmoscopically in one of the dogs could not be identified on MRI.

## Measurements

Results of the descriptive statistics for the variables measured with HR-MRI and US, and of the Wilcoxon rank sum test for the comparison of both modalities (with the exception of the optic nerve diameter, measured in MRI only) are presented in Table 3.

**Table 3:** Summary of ocular measurements obtained via MRI and US in 10 Beagle dogs

Parameter	Modality	Median	Range	SD	Min	Max
CCT	US	0.90	0.02	0.065	0.80	1.00
	MRI	0.65	0.02	0.075	0.57	0.81
ACD	US	4.10	0.14	0.451	3.39	4.70
	MRI	4.31	0.08	0.245	3.99	4.64
AQD	US	3.12	0.13	0.419	2.51	3.80
	MRI	3.61	0.08	0.263	3.13	3.99
APLD	US	7.58	0.11	0.350	7.14	8.40
	MRI	8.23	0.05	0.166	8.05	8.55
MLLD	US	12.11	0.15	0.465	11.40	12.90
	MRI	12.46	0.06	0.184	12.20	12.80
DVLD	US	12.18	0.13	0.408	11.50	13.00
	MRI	12.43	0.06	0.183	12.10	12.70
VCD	US	9.23	0.18	0.566	7.83	9.87
	MRI	9.56	0.08	0.254	9.17	9.88
APDSRR	US	22.26	0.15	0.474	21.30	22.90
	MRI	23.34	0.09	0.280	23.00	23.80
APD	US	20.87	0.18	0.577	19.90	21.80
	MRI	22.18	0.11	0.355	21.70	22.70
ONSD	US	2.68	0.04	0.137	2.40	2.87
	MRI	2.00	0.03	0.102	1.87	2.14
OND	MRI	1.82	0.02	0.068	1.74	1.98

**Fig. 8:**
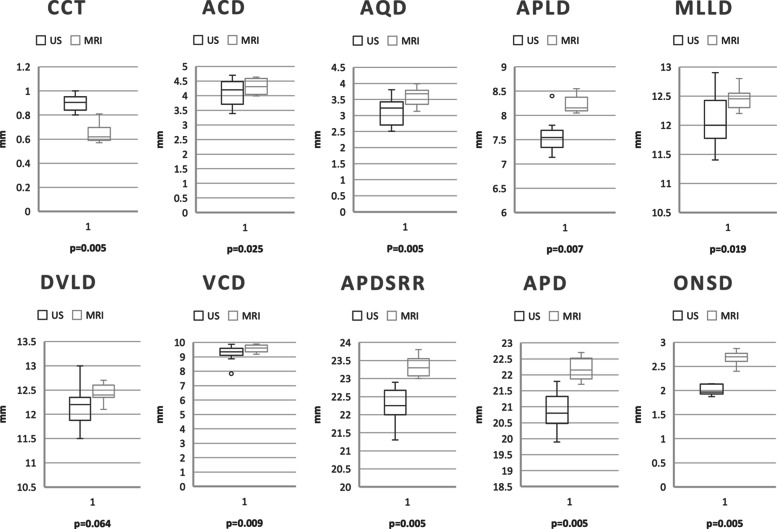
Distribution of measurements in US and HR-MRI. Box plots representing the distribution of the measurements in US and HR-MRI. The whiskers represent minimum and maximum values, *p* values

All HR-MRI measurements were significantly different from US, with the exception of DVDL, which fell slightly above the significance level. The respective *p* values are summarized in Fig. 8. No statistically significant differences between left and right eye HR-MRI measurements were found. The diameter of the optic nerve (OND) was only determined in MRI and measured 1.82 ±0.07mm (Table 3).

## Discussion

The use of 3T HR-MRI in conjunction with micro-coils provided excellent signal-to-noise ratio and exquisite anatomical detail of the bulbus oculi and orbital structures in dogs due to the sub-millimeter resolution. MRI studies achieving resolutions of less than 100 *μ*m are sometimes referred to as MRI-biomicroscopy; this could be achieved in our study using a commercially available 3T HR-MRI machine in conjunction with experimental surface coils. Due to its tomographic nature, slice positioning and acquisition of images in the desired anatomical planes was accurate and not as technically challenging in HR-MRI as in US or UBM. Overall, the HR-MRI images were considered superior to US and UBM, and identification and distinction of structures not previously described with MRI was possible. HR-MRI allowed assessment of all structures identified with US and UBM.

HR-MRI, US and UBM all have their specific advantages and limitations. The MRI examinations were performed in general anesthesia with the addition of a neuromuscular blocking agent (NMBA), while US and UBM examinations could be performed in awake animals, with only the use of topical anesthesia. An NMBA was necessary and successfully used to eliminate globe movement under general anaesthesia, with additional injections given every 20 minutes for the length of the procedure (2-3 times per procedure). The duration of the HR-MRI examination ranged between 40-50 minutes per eye in our study, but this can be significantly reduced by optimizing the imaging protocol for specific clinical settings. Alignment of the imaging planes to the axis of the bulbus oculi could be successfully achieved in all dogs in HR-MRI but was dependent on the compliance of the dog in US since all US and UBM examinations were performed in awake animals. Also, canine head and nose anatomy can limit US access to the ventral and nasal aspects of the globe, making access virtually impossible with UBM in awake animals. UBM is limited by the inherent shallow tissue penetration of 5 to 10 mm which limits the field of view to the anterior segment [30]. Further limitations of US include the fact that resolution (lateral and axial) is affected by imaging depth [31] and that structures at the boundary of the globe cause non-perpendicular reflection of the ultrasound beam, translated into distal hypointense streaks [31], which can hinder examination of off-axis structures. UBM can achieve a lateral resolution of about 20-50 *μ*m [32], the lateral resolution of the US probe used in our study was calculated at approx. 290 *μ*m and HR-MRI achieved an in plane resolution as low as 46x46 *μ*m. Based on these theoretical resolutions and also based on our observations, HR-MRI and UBM have a similar resolution, which is clearly higher than the resolution afforded by conventional US.

While the requirement for general anesthesia with use of an NMBA is a limiting factor for HR-MRI, we consider that the benefits can outweigh the risks in selected cases, where high resolution imaging of structures is required that cannot be reached with UBM. Modalities not used in this study, such as optical coherence tomography superior resolution but can be limited by ocular opacities and lesion localization.

Higher doses of neuromuscular blocking agents (NMBAs) can provoke transient respiratory depression and diaphragm paralysis, which require mechanical ventilation for the duration of the effects [20]. This increases complexity of the anesthetic protocol and marginally increases overall costs. The possibility to administer an NMBA that produces only the blockade of the extraocular muscles, without significantly affecting the respiratory function, could be beneficial [20], however, to our knowledge, this has not yet been achieved. Assessment of the cornea and sclera was considered superior with HR-MRI compared to US and UBM, as the entire circumference of the globe could be displayed at high resolution. The trilaminar appearance of the canine cornea in MRI images in fluid-sensitive sequences such as T2w and T2SPAIR was not previously described, to our knowledge. We speculate that this could be attributed to the presence of highly aligned collagen fibers within the stroma, magic angle or chemical shift artefacts. The MRI appearance of collagenous tissues in other regions of the body has been shown to be strongly influenced by the anisotropic arrangement of collagen fibers, preferential alignment of water molecules associated with collagen and by the alignment of the specimen relative to the magnetic field [33]. The use of 9.4T MRI on formalin-fixed ovine corneoscleral tissues revealed microstructural details of the cornea and sclera and changes therein with intra-ocular pressure elevation (IOP) [14]. Differentiation of the sclera, choroid, and retina with MRI was previously reported to be impossible in dogs [34]. However, a multi-laminar structure of retina and choroid was described in the eyes of mice [35], rats [36], cats [37], baboons [12] and humans [38, 39] using high field MRI. The imaging features of the three discrete, distinct, laminar structures appearing as alternating hyper-, hypo-, and hyperintense bands that were identified in the area of the choroid and retina in our study were consistent with those described in other species. Previous studies correlated the innermost T1 and T2 hyperintense layer with the nerve fiber, ganglion cell, inner plexiform, inner nuclear and outer plexiform layers and their embedded retinal vasculature. The middle T1 and T2 hypointense layer correlates with the avascular photoreceptor cell layers, including the outer nuclear layer and photoreceptor inner and outer segments within the outer retina. The outermost T1 and T2 hyperintense layer, with marked contrast uptake, was assigned to the tapetum and choroidal vascular layers [12, 35–38]. We present the first description of the distinction of the avascular photoreceptor layers of the retina from the vascular inner retina and choroid in dogs, using a clinical 3T MRI scanner with surface micro-coils. Detailed images of the anterior and posterior uvea could be acquired with HR-MRI, revealing anatomical details previously not discernible in MRI studies of the canine eye, such as the ciliary cleft and ciliary processes. The T1 hyperintense appearance of the ciliary body and posterior layer of the iris was likely caused by the presence of melanin known to provide a high T1 signal intensity [40] in the heavily pigmented outer ciliary and posterior iris epithelium, and by contrast provided by the hypointense background of the anterior and posterior chambers and vitreous. Assessment of the ciliary cleft was similar or superior in dorsal or sagittal plane T2w and T2SPAIR HR-MRI images compared to UBM. Due to the fact that the ciliary body was ill-defined and provided little contrast in the sagittal and dorsal HR-MRI planes, UBM was considered superior to HR-MRI when examining the ciliary body in these planes. Delineation of the ciliary body was improved in T1w post-contrast images, profiting from the marked enhancement of vascularized structures and the low background signal of the posterior chamber and vitreous. Both HR-MRI and UBM provided excellent transverse images of the ciliary body, which were superior to US. HR-MRI presented the advantage of allowing visualization of the entire Corona ciliaris (in 9 out of 10 dogs) and the anatomy posterior to it, thus overcoming the technical limitations of US and UBM, such as resolution, positioning, acoustic shadowing, refraction and reflection artefacts, limited acoustic window and limited penetration – the latter representing more of an issue in UBM. The combination of limited access to the ventral and nasal aspects of the globe, and limited tissue penetration make UBM examination of the ciliary body outside the dorsal and temporal quadrants extremely challenging in awake animals. HR-MRI was superior to US for examination of the lens, due to the fact that it allowed delineation of the entire anterior lens capsule and posterior contour of the lens, while US is commonly affected by artefacts towards the equators of the lens in sagittal and dorsal images. The area of the optic disc, Tenon’s capsule and the sub-Tenon’s space could only be identified with HR-MRI. In fact, we present a more detailed depiction of Tenon’s capsule and the sub-Tenon’s space than described in the current literature. Direct depiction of the sub-Tenon’s space with HR-MRI opens up possibilities for future research into the application of sub-Tenon’s medications or anesthesia, which is the most widely employed regional periocular block in many countries [41, 42]. HR-MRI allowed excellent depiction of the optic nerve within its sheath, especially in fat-saturated images (T2 SPAIR). This was possible in all anatomical planes, but the extent to which the nerve could be examined was limited to the intraorbital region, due to the inherent small field of view provided by the micro-coils and low signal from the intracanalicular region. ONSD values, which have previously been established as a marker for intra-cranial pressure [43], provided by US were significantly lower than those acquired via HR-MRI. We measured a mean ONSD of 2.7 mm (range: 2.4-2.87 mm) via HR-MRI in Beagle dogs. ONSD can vary with breed, as shown in Yorkshire terrier and Maltese dogs by Lee et al. [43]. The diameter of the optic nerve (OND) was 1.81 mm (range: 1.74-1.98 mm), which is in agreement with previously published values(18). The authors note a correspondence paper by Copetti and Cattarosi [44] which highlights possible errors due to acoustic shadowing in US-derived optic nerve sheath diameter measurements in humans and acknowledge that this may also apply to veterinary practice. HR-MRI provided excellent anatomical details of the retrobulbar region, allowing assessment of all extraocular muscles and the majority of the adnexa of the globe. The mean values for AQD, ACD, DVLD, MLLD, APLD, VCD, APDSRR and APD were higher and the measurement range was narrower for HR-MRI than for US. This could be due to differences in resolution, caliper placement accuracy and potential obliquity of acquired US images, causing measurements to not be determined along the central axis of the globe, which would increase measurement variability. Also, gentle pressure as a result of US probe placement can cause subtle globe deformation resulting in decreased ACD, AQD, VCD, APDSRR and APD values. The lower average CCT values via HR-MRI compared to US could be due to more exact placement of calipers, aided by the higher resolution and clearer delineation of the cornea in MRI.

## Conclusions

The findings from this prospective in-vivo study prove the feasibility of the use of 3T HR-MRI in conjunction with micro-coils to depict the canine eye in great detail. Visibility of the entire ocular wall, the lens, structures caudal to the ciliary body and the optic nerve and its sheath was superior with HR-MRI compared to regular US. HR-MRI allowed the distinction of the retina, choroid and sclera, and the delineation of structures not previously identified in canine eyes with MRI, including Tenon’s capsule and the sub-Tenon’s space.

The use of general anesthesia with the addition of a neuromuscular blocking agent provided adequate reduction of motion artefacts for HR-MRI, while US and UBM were performed with the animals awake.

While not replacing conventional modalities, HR-MRI may be useful for future research applications and for clinical assessment of ocular pathologies as it becomes more widely available in veterinary practice.

## Data Availability

The complete raw and analysed datasets are available from the corresponding author on request.

## Abbreviations

HR-MRI: high-resolution magnetic resonance imaging
US: ultrasound
UBM: ultrasound biomicroscopy
DVLD: Dorsoventral Lens Diameter
CCT: Central Corneal Thickness
AQD: Aqueous Depth
APLD: Anteroposterior Lens Diameter
VCD: Vitreous Chamber Depth
ACD: Anterior Chamber Depth
APD: Anteroposterior Depth of the eye excluding the scleroretinal Rim
APDSRR: Anteroposterior Depth of the eye including the Scleroretinal Rim
MLLD: Mediolateral Lens Diameter
ONSD: Optic Nerve Sheath Diameter
OND: Optic Nerve Diameter
